# Bounding the costs of electric vehicle managed charging—supply curves for scenarios from 2025 to 2050

**DOI:** 10.1038/s41597-026-07008-6

**Published:** 2026-03-11

**Authors:** Reiko Matsuda-Dunn, Elaine Hale, Ellie Estreich, Luke Lavin, Gabriel Konar-Steenberg

**Affiliations:** Grid Planning and Analysis Center, National Laboratory of the Rockies, 15013 Denver West Parkway, Golden, 80401 CO USA

**Keywords:** Energy modelling, Energy infrastructure

## Abstract

As electric vehicle (EV) adoption increases, the resulting EV battery charging will increase demand on the electric power grid. Through EV managed charging (EVMC) programs, charging can be shifted in time to support electric grid reliability and reduce electricity costs. EVMC can offer an alternative to additional supply-side generation, but the costs of EVMC implementation must be understood to evaluate the cost-benefits of EVMC. This paper presents bottom-up, forward-looking (from 2025 through 2050) estimates of the incremental costs associated with different EVMC dispatch mechanisms available to electric utilities. The costs of enabling EVMC for a range of customer participation levels are presented in the form of supply curves, which provide per-EV costs for a targeted level of participation. The largest drivers of cost variation are assumptions about future charging flexibility paradigms described in four scenarios. These supply curves can be used to quantify the expected costs of EVMC programs and enable comparison with supply-side or other demand flexibility alternatives.

## Background & Summary

There are more than 4.8 million electric vehicles (EVs) estimated on the road in the United States^1^, and the International Energy Agency’s Global EV Outlook 2024 report estimates this figure will grow to 30 to 42 million by 2030^2^. Globally, the number of EVs has also been growing at an increasing rate over the past decade^3^. EV adoption increases electricity demand, which can require additional electricity generation and trigger transmission or distribution system capacity upgrades.

However, strategies such as managing the timing of EV charging can delay or avoid capacity upgrades. This enables more near-term load growth at less cost by alleviating peak demands at various points in the power system. Further, these strategies can make better use of lower-cost generation by reducing system peak net load and reducing net-load variability^4^. In either case, EV managed charging (EVMC) supplies a grid service and is cost effective as long as EVMC enablement costs are less than the costs of providing the same service with a supply-side resource (e.g., generator, transmission upgrade, or distribution upgrade) on a per-kilowatt (kW) or per-kilowatt-hour (kWh) basis.

There are different ways to manage EV charging across a participating fleet of EVs, which are reflected in different types of utility programs. This article describes a dataset of estimated EVMC costs for three program types: direct load control (DLC), time-of-use (TOU) tariffs, and real-time pricing (RTP). Each of these program types have different value propositions in terms of value per participating vehicle at different levels of participation^5^. How these program types (or dispatch mechanisms) operate and how they are perceived by potential participants differ, which has implications for how different categories of EVMC costs evolve over time, how many people might be willing to participate, and what incentive levels are required to elicit that participation.

- **Direct Load Control** DLC programs allow utilities or aggregators to schedule or modulate vehicle charging remotely, without vehicle owner actions aside from selecting initial settings such as expected departure time, minimum state of charge at departure, or acceptable cost. Programs using dynamic dispatch of vehicle charging tend to rely on two-way communication to directly coordinate EV charging loads with grid capacity and energy supply. This can occur either via the charger or through on-board vehicle telematics. DLC provides the most control of a given demand response resource, but literature has shown lower enrollment rates because of a portion of eligible customers being averse to utility or aggregator control of their vehicle^6^. Dependence on frequent communication can also result in reduced participation resulting from communication reliability challenges. Subject matter experts cited account login changes, firmware updates, WiFi not reaching a customer’s garage, or other technical issues as common reasons for nonparticipation^7,8^.
- **Real-Time Pricing** RTP programs use dynamic price signals sent to customers that reflect wholesale prices on a day-ahead or week-ahead basis, for example. Similar to DLC, RTP programs identified for EVMC rely on frequent communications with vehicles, vehicle owners, or chargers. They can therefore also be impacted by the communication reliability challenges described previously for DLC programs. However, communications to support RTP may not require the same data throughput for successful participation as DLC, as price signals might only be sent once a day, and vehicle charging might be directly metered on-site or only communicated to the program operator once a week or once a month, for billing purposes. Nonetheless, lacking specific data on how costs might differ, we assume that RTP operating and administrative costs are the same as those of DLC. Modeled RTP and DLC costs differ overall due to customer incentives and participation. Some degree of customer aversion to RTP enrollment is anticipated, but RTP is assumed to be acceptable to more customers than DLC because scheduling is more transparent and directly controlled by the driver/owner.
- **Time-of-Use** TOU programs financially incentivize customers to charge at preferable times of day through a static, time-based rate structure that includes lower- and higher-priced time blocks. In the Low-Flexibility scenario, TOU is assumed to be a largely manual program, in which software to program EV charging to automatically occur during lower-priced times is not necessarily available, but participants can manually choose to plug in their EVs at off-peak times. In other scenarios, continued software rollout enables widespread automation. TOU is still a static, time-based rate structure, but customers avoid on-peak charging programmatically, thereby increasing the effective participation rate by pushing more charging to off-peak periods because manual intervention from vehicle owners is not needed^9^.

These three program types were selected because they are (1) the commonly found or proposed and (2) are distinct from one another. Vehicle to grid, or V2G, programs that involve bidirectional power flow are more nascent, but may have a very different value proposition. V2G is excluded from this work. EVMC programs of all types are growing in number, yet there is a gap in understanding how the share of EV owners participating in EVMC programs—or the “supply” of flexible EV loads—is related to program costs, as well as how the relationship between costs and participation might change under future conditions. The relationships between EVMC participation rates, incentives, and implementation costs have implications for evaluating the cost-effectiveness of EVMC and its contributions to supporting grid reliability and affordability. Anwar *et al*. (2022) review a range of studies on EVs and describe this data gap by noting that more complete benefit-cost assessments “consider[ing] the entire extent of values, enablement costs, and the perspectives of all stakeholders” are often missing, indicating the need for data that describe the resource potential of EVMC weighed against the comprehensive costs of program administration and customer participation^4^.

Previous work has developed estimates of the costs and potential quantities of EVMC. DR-Path, a model developed by Lawrence Berkeley National Laboratory, generates demand response supply curves comprising shed and shift capabilities and costs for many flexible electric end uses, including EVMC. DR-Path represents a variety of demand response programs including dynamic price signaling and TOU, and conceptualizes EVs as a demand shifting resource that responds to a dispatch signal such as a price. The resource potential is assembled from cost and resource availability data, including incentive-dependent enrollment propensities, that vary with site-specific attributes and customer demographics^10^. Although detailed and informative, the underlying data and results are California-specific and are also not packaged or normalized for easy reuse in other contexts. Academic studies and consulting reports have also published data on pilot programs^6,8,9^ or made informed assumptions about program costs and participation rates associated with EVMC programs^11,12^. However, with the exception of DR-Path, none of these studies are forward-looking or consider temporal changes in the relationship between incentives and EVMC program participation. In addition, although DR-Path and studies by Guidehouse^8^ and the Brattle Group^12^ consider multiple cost categories, none of the previously cited studies consider how these costs vary across multiple EVMC program types and cost structures.

Wong *et al*.^6^ distribute a survey across the United States and use mixed logit models to understand which factors impact participation in EVMC programs; however, their estimates are constrained to a single time period. None of the other studies are nationally representative and rather rely on geographically specific pilot programs or older market data and/or assumptions about key parameters, limiting their usefulness and application to broader decision-making contexts.

This work extends previous research by developing EVMC supply curves—functions characterizing the quantity of an available resource in relation to the cost of the resource—that estimate the per-vehicle costs of participation in EVMC at specified adoption levels from 2025 through 2050; 2025 USD are used in all cases. The authors use a forward-looking, scenario-based framework and generate individual supply curves for EVMC across four different scenarios and according to vehicle type (light duty or medium and heavy duty), customer type (new or recurring), and program type (DLC, TOU, or RTP), and for 5-year increments from 2025 to 2050. These four scenarios reflect different assumptions about the flexibility potential of EV loads and to what extent EV charging and program design will change, thereby affecting access to programs, enrollment, and participation. Our study is the only one we are aware of that estimates EVMC supply curves using a scenario-based framework to handle future uncertainty, which broadens our analysis in a way that may be useful for decision makers contemplating different possible futures.

**Fig. 1:**
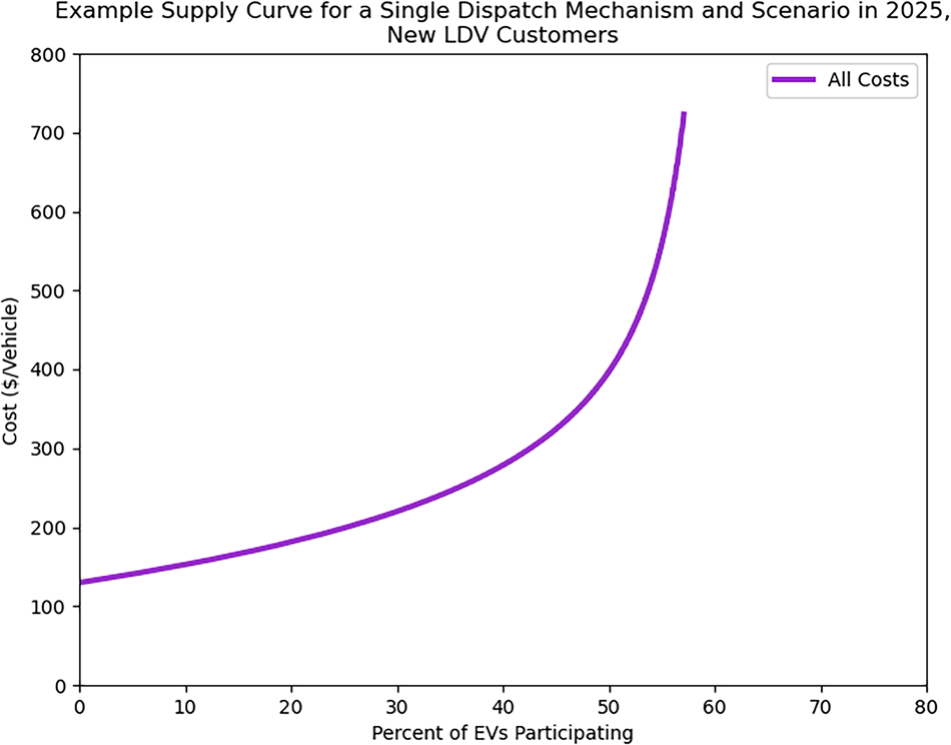
Example supply curve for the costs of new customer enrollment per vehicle. This curve is produced for DLC customers in a mid-range scenario. In this instance, costs of increasing participation in EVMC largely result from requiring increasing customer incentive for participation. All participation requires incurring fixed costs for marketing, administration, and operating the EVMC program.

The authors identify the most recent cost and participation data for EVMC programs from a variety of sources, including academic literature, surveys, utility filings, and interviews (these are enumerated in Table 9), then calibrate these data to match conditions under each proposed scenario and project out to 2050. This work takes a more comprehensive view of program costs than prior studies by identifying costs across several categories, including marketing, program operation, administration, and customer incentives. The authors then apply a set of additional assumptions about the nature of demand response program participation to estimate the shape of the relationship between program costs and participation. An example supply curve is shown in Fig. 1. This example is for new customers with light-duty vehicles (LDVs) enrolled in a DLC program in 2025. In the example curve shown, fixed annual costs per vehicle include initial administration cost, program operating costs, and marketing costs. Variable costs include incentives, which increase customer enrollment.

Individual supply curves for 2030 and beyond rely more heavily on assumptions about how costs will develop. Yet these forward-looking curves can potentially benefit utilities, grid planners, and others seeking to understand how EVMC could scale and be part of planning and operating the future electric grid. Forward-looking supply curves may also benefit assessments of the anticipated impact of EVMC programs on power system outcomes such as electric demand and peak load. In general, we anticipate the presented supply curves will be relevant to many grid stakeholders—including electric utilities, research institutions, industry stakeholders, aggregators, regulators, power system operators, and equipment suppliers—for a variety of use cases and applications. These curves are constructed primarily from data sources throughout the United States and are not tailored to specific regions. Varying policy environments, EV adoption levels, availability of enabling technology, and customer characteristics will influence the appropriate supply curve selections and applications. Users can examine scenario assumptions and case-specific ranges for cost categories to select supply curves to best represent EVMC in each use case.

## Methods

The authors assemble cost and participation data for EVMC pilots and programs found in utility filings, reports, prior research, and subject matter expert interviews into a structured supply curve format that relates cost to participation rate. The data sources and research findings used to quantify supply curve points and support various modeling assumptions are systematically documented in Technical Validation section (especially Table 9) and are also cited in context in the remainder of the article. Most data sources focus on LDV EVMC programs. For the more nascent medium- and heavy-duty (M/HDV) EVMC programs, we rely heavily on M/HDV program costs reported by National Grid, as few data points were found elsewhere^13^. It is important to keep in mind that the data only describe the costs of EVMC and do not include electric vehicle supply equipment (EVSE) or other costs that might be incurred to more broadly enable EV adoption. That is, these supply curves assume the EVSE and other infrastructure required to support EV adoption are already in place. They then describe the *incremental* costs required to better align EV charging with available grid capacity and generation through EVMC TOU, RTP, or DLC programs.

**Fig. 2:**
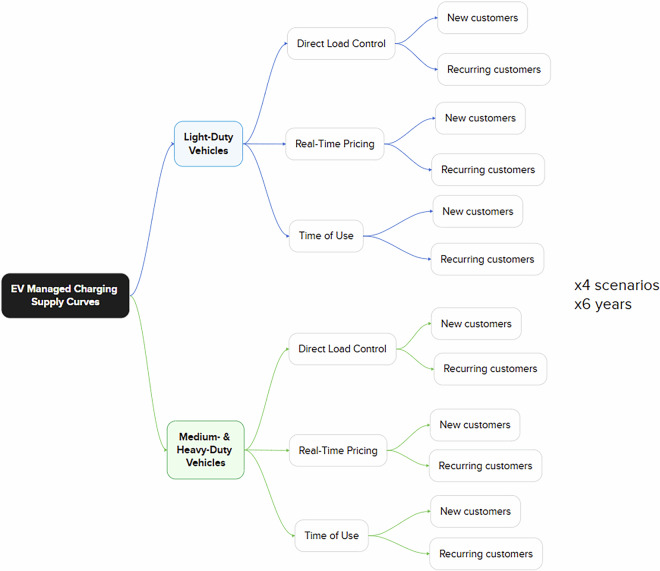
Twenty-four sets of supply curves are generated for four different scenarios (Flat, Low, Mid, and High Flexibility) and six different years (2025, 2030, 2035, 2040, 2045, and 2050). Within these sets, individual supply curves reflect differences between two vehicle types (LDV and M/HDV), between three program types (DLC, RTP, and TOU), and between new and recurring customers. Altogether, this results in 288 final curves although some are identical, because there are no cost differences between new and recurring M/HDV TOU participants in three of the four scenarios and TOU LDV costs are identical across the High and Flat scenarios. Accounting for these duplications results in a total of 234 unique supply curves.

Future costs are projected in 5-year increments through 2050 for four scenarios driven by plausible narratives of how EVMC technologies and costs might evolve over time. Within the 24 resulting sets of supply curves (one for each scenario and year), costs vary by vehicle type (LDV and M/HDV), program type (DLC, RTP, and TOU), and new or recurring customers (Fig. 2). Many cost differences are driven by the different program types, their implementation requirements, the way they enroll or otherwise engage customers, and their operation method. We provide four different projections to bound potential changes in EVMC program costs and to enable planners to select the trajectories that seem most relevant in their contexts. These scenarios are defined and described in the Methods section. To preview, they represent different cases in which EV charging flexibility is easier (less expensive) to access (High Flexibility), harder (more expensive) to access (Low Flexibility), a mid case (Mid Flexibility), and a scenario that encourages spreading EV charging over more time at lower power levels to produce “flattened” charging profiles (Flat) is motivated. All supply curves define costs across similar categories, although interpretations differ somewhat, depending on program type and new or recurring customers. The cost categories, their meaning in different contexts, and their definition in functional forms and assumed parameter values are included in the Methods subsection, Cost Categories. The assembly of data across all categories into supply curves is described in another Methods subsection, Supply Curve Formulation.

Across the dataset’s 4 scenarios, 6 years (2025, 2030, 2035, 2040, 2045, and 2050), 2 vehicle types, 3 program types, and 2 customer types (new or recurring), there is a potential for 288 different EVMC supply curves. However, there are two instances in which we assume the same costs across some variables, which reduces the unique supply curve count by 54, to a total of 234. First, in most cases, we assume that new and recurring customer costs are different either because there are one-time, initial administrative costs associated with signing up a new participant, or the first-year participation incentive is different (higher) for customers requiring a new charger to participate, or both. However, in three of the four scenarios, we assume the costs for M/HDVs participating in TOU programs are the same for new and recurring customers, based on the assumption that no added metering or other hardware, nor any additional administrative actions, are needed for new customers^13^. Second, for LDVs, the supply curves for RTP and DLC in the Flat scenario are the same as the Mid-Flexibility scenario, as the level of fast charger adoption (versus charging at L1) is not expected to influence the cost of or maximum level of participation in managed charging programs (although some programs might use the available charging level as a screening criterion for eligibility). For LDVs, the supply curves for TOU in the Flat scenario are the same as the High-Flexibility scenario, as the Flat scenario aligns with the adoption of enabling technology that increases participant adherence to On-Peak and Off-Peak periods.

### Scenarios

EVMC supply curves are designed to align with different scenario concepts to facilitate the examination of EVMC’s impact on bulk power system planning and operation under different plausible descriptions of how EVMC costs and willingness to participate might evolve over time or be expressed in different locations. Three scenarios provide low-, mid-, and high-flexibility assumptions, whereas a fourth, flat, scenario anticipates increased daytime EV charging access and prioritization of lower-power EV chargers in homes (for LDVs) and at depots (for M/HDVs) to encourage flatter EV loads. For many of the cost categories described in Section, a range of probable costs were identified. These are expressed across the different scenarios based on the scenario narratives.

- The** Mid-Flexibility** scenario represents midline cost assumptions. Managed charging program implementation benefits from increased enabling technologies, customer education, and program operation efficiencies. These data could be paired with midline EVSE access and use assumptions that start from existing consumer behaviors that express preference for home (LDV) and depot (M/HDV) charging and then project forward-declining proportions of charging at such locations as the market moves from early adopters to mass market consumers and/or some EVSE deployments are designed to encourage more daytime charging.
- The** High-Flexibility** scenario posits plentiful, highly capable chargers and breakthroughs in controls and communications that result in lower costs, more EVMC participation mechanisms, and more EVMC participation by default. Lower costs include not only increased customer access to smart chargers (or fewer customers that would need to invest in new chargers to participate) but also lower administrative, marketing, and incentive costs, which could occur if scheduled charging became routine for drivers and utilities alike.
- The** Low-Flexibility** scenario provides an upper bound to the costs of EVMC. Enabling technology does not proliferate as readily as in the other scenarios, and customer access to smart charging is lower. Limitations to access to reliable, smart charging opportunities result in fewer EVMC participants in DLC and RTP programs, with greater incentives required to motivate customer response. TOU participation does not benefit from programmable charging. These data could be paired with EVSE deployment assumptions that maximize convenience over coordination with energy supply to provide an upper bound on the grid costs of EV integration.
- The** Flat** scenario is aligned with concerns around the distribution and transmission upgrades that could be needed to support many high-powered chargers and the potential for their simultaneous use^14^. Concerns that distribution or transmission system capacity could be exceeded are passively mitigated in the Flat scenario by prioritizing the installation of lower-powered chargers at depots, public locations, or other eligible areas, and by motivating slower, flatter EV charging. Flattening EV load and decreasing potential for peaks from high-power chargers mitigate concerns about simultaneous-charging use consumption spikes. We assume that, for LDVs, such a scenario would manifest as fewer L2 and higher-powered chargers. The LDV TOU supply curves available in this dataset are thus the same for the High-Flexibility and Flat scenarios, whereas supply curves for LDV RTP and DLC programs are the same for Low-Flexibility and Flat scenarios. M/HDVs are assumed to require higher incentives to participate in EVMC programs relative to the Mid-Flexibility scenario, because the default charging mode is already assumed to be lower powered and thus flatter.

### Assumptions

The supply curves are constructed with data and resources supporting cost category-specific bounds, presented in Table 9. In cases of sparse or wide-ranging data, there are assumptions that can be made that inform supply curve construction. These assumptions underpinning supply curve formulation are documented here:

- The shape of enrollment response curves to incentives is a decaying exponential approaching an asymptote. This is demonstrated by Wong *et al*.^6^ and used in prior work^15^.
- M/HDV programs are informed by few data sources, primarily National Grid’s reported costs^13^.
- EVMC programs do not include the cost of EVSE and costs that occur regardless of EV owners participation in EVMC. EVSE is already installed or will be installed regardless.
- Future costs are reasonably bounded by the four scenarios described above to capture a range of drivers for EVMC flexibility.
- New participants will typically cost more than recurring participants, with the exception of M/HDVs in TOU programs, as they will already be metered (no new installations).

### Cost Categories

To represent EVMC enablement costs, the authors develop initial data and forward-looking assumptions for four different cost categories: initial administrative costs, program operating costs, incentive costs, and marketing costs. The costs reflect only managed charging enablement costs experienced by the utility. They do not include EVSE or other costs (e.g., panel upgrades, distribution system upgrades) expected to co-occur with EV adoption, whether or not owners participate in a managed charging program. However, we do account for the utility cost share (usually disbursed in the form of a rebate) for new chargers that some LDV owners might need to purchase to participate in DLC or RTP programs that require frequent communication.

For recurring participants, costs consist of program operating costs, marketing, and annual incentives. Signing up new participants is sometimes more costly on a per-vehicle basis, requiring an initial administrative cost and/or an initial incentive (to support the purchase of a new charger) that is higher than the annual incentive provided to already-enrolled participants.

#### Initial Administrative and Program Operating Costs

Annual administrative and program operating costs for new LDV participants in an EVMC program are reported in utility documents and prior research^15,16^. Interviews with subject matter experts indicate additional fees from automakers could increase this amount to an upper estimate that is included in Low-Flexibility scenario assumptions. RTP and DLC costs are similar: while communication mechanisms may vary, DLC will require more timely but less frequent dispatches than RTP. Similar costs were observed in the literature^15,16^. Initial administrative costs for TOU are lower in all cases except for the Low-Flexibility scenario. Initial administrative and operating costs are higher for M/HDV vehicles, with figures from utility filings and prior work^13,15^. In Flat, Mid-, and High-Flexibility scenarios, TOU has no additional initial costs, as some utility programs report annual administrative and program costs are the same for new and recurring participants.

The dataset’s initial administrative costs by vehicle type, program type, and scenario are shown in Table 1. New customer administrative and program operating costs are equal to these costs plus the program operating costs for recurring customers reported in Table 2. Initial administrative costs are assumed constant over time, while the program operating costs generally decline. The result is that overall administrative and program operating costs decline over time for both new and recurring participants.

**Table 1:** Initial administrative costs for all vehicle types, program types, and scenarios.

Vehicle Type	Program Type(s)	Scenario(s)	Initial Administrative Cost ($/vehicle)
LDV	DLC & RTP	Mid-Flex & High-Flex	60
LDV	DLC & RTP	Low-Flex & Flat	120
LDV	TOU	Mid-Flex	50
LDV	TOU	High-Flex & Flat	10
LDV	TOU	Low-Flex	120
M/HDV	DLC & RTP	All	250
M/HDV	TOU	Mid-Flex, High-Flex & Flat	0
M/HDV	TOU	Low-Flex	120

Costs are assumed constant over time.

**Table 2:** Program operating costs that apply to both new and recurring EVMC participants.

Vehicle Type	Program Type(s)	Scenario(s)	2025	2030	2035	2040	2045	2050
LDV	DLC & RTP	Mid-Flex & Flat	60	55	50	45	42	40
LDV	DLC & RTP	High-Flex	60	50	40	30	25	20
LDV	DLC & RTP	Low-Flex	120	105	90	80	70	60
LDV	TOU	Mid-Flex & Flat	60	55	50	45	42	40
LDV	TOU	High-Flex	50	40	30	25	20	20
LDV	TOU	Low-Flex	0	0	0	0	0	0
M/HDV	DLC & RTP	Mid-Flex & Flat	250	150	100	90	70	60
M/HDV	DLC & RTP	High-Flex	250	100	80	70	60	50
M/HDV	DLC & RTP	Low-Flex	250	250	250	250	250	250
M/HDV	TOU	Mid-Flex & Low-Flex	250	150	100	90	70	60
M/HDV	TOU	High-Flex & Flat	250	100	80	70	60	50

Values listed per year are in 2025-$ per participating vehicle.

#### Program Operating Costs

Annual operating costs for recurring customers are reported in utility program filings and were confirmed in subject matter expert interviews^13,16^. Operating costs per customer are assumed to decrease over time as operations become streamlined and more customers enroll. There are two exceptions to this, both in the Low-Flexibility scenario: (1) Low-Flexibility M/HDV DLC and RTP program operating costs are modeled such that no per-vehicle cost savings occur over time, which is indicated in some utility forecasts, and (2) Low-Flexibility TOU programs for LDVs are conceptualized as low-tech implementations with no software support, reducing customer adherence to off-peak charging but also requiring zero program-operating costs. This might occur, for example, in a utility with a general TOU rate that applies to all consumption, not just EV charging, such that there is no additional cost specifically for EVMC.

#### Incentives

Utilities often provide incentives for customers to enroll in DLC or RTP programs, and the literature observes both increasing levels of participation with increasing incentives and a portion of the population will not participate for any incentive^6^. The functional relationship of participation rate versus incentive level generally has a declining slope, that is, increased incentive levels have a strong effect near the origin of no incentive and no participation, but then the slope declines. As the slope declines, the curve flattens out toward an asymptote of maximum participation as incentive levels exceed what is required to entice most potential participants.

To capture this relationship, we model participation rate *r* as a function of incentive level *x* using an exponential decay function:

1 $$r=\overline{r}\cdot \left(1-{e}^{-\beta \cdot x}\right),$$

where the participation rate *r* is a number between 0 and 1, $$\overline{r}$$ is the maximum participation rate, *x* is the incentive level in 2025-$ per vehicle per year, and *β* is a model parameter that defines the shape of the curve. If there were numerous data points available, $$\overline{r}$$ and *β* could be estimated to provide the best fit. However, the actual data are fairly sparse. We calculate *β* after specifying $$\overline{r}$$ and a point $$\left({x}^{\ast },{r}^{\ast }\right)$$ assumed to be on the curve: that is, we calculate *β* as:

2 $$\beta =-\frac{1}{{x}^{\ast }}\cdot ln\left(1-\frac{{r}^{\ast }}{\overline{r}}\right)$$

for different vehicle types, program types, scenarios, and years. The response curves could also be different for new and recurring customers. However, to reduce the number of assumed values, we hold $\overline{r}$ constant across all new and recurring customers and assume incentive levels only have to be different (higher) for new customers that require a new smart charger to participate, which we assume is limited to a fraction of LDV owners considering participation in a DLC program.

Although thus far we have only mentioned modeling participation rates for DLC and RTP programs, as a function of incentive level, TOU programs also have varying levels of adherence (i.e., the amount of charging load actively scheduled to avoid higher-priced peak times). For M/HDVs, we assume there is an incentive for participating in TOU, just as there is for DLC and RTP, and model participation rates as described previously. However, LDV TOU programs are assumed to have no incentives for participation, and we instead model the level of adherence as a function of marketing costs, which are assumed to correlate with customer awareness of TOU rates and how to ensure their cars or chargers avoid charging during higher-priced times. In this case, the functional form is the same (Equation (1)), but *x* and x^*^ are marketing costs in 2025-$ per vehicle-year rather than an annual incentive, and *r* is interpreted as an adherence rate.

In setting maximum participation rates ($\overline{r}$), we observe that surveys, pilot data, and interviews indicate a portion of EVs will not enroll in a program or respond to a retail rate signal at all, because of customer aversion, or will be unavailable for a portion of the time because of communication limitations such as firmware updates or communication network access issues. This unavailability is represented as an upper limit on participation $\overline{r}$ that is strictly less than 1, whose values we estimated from survey^6,17,18^ and pilot^8,9,19,20^ data and are shown per program type and year in Table 3. These limits are assumed to be the same across LDVs and M/HDVs and for new and recurring participants. DLC and RTP upper limits are different for each program, with DLC upper limits 10% lower than those of RTP programs because of greater customer aversion to the utility or a third-party scheduling or modulating EV charging. This results in more expensive per-vehicle costs associated with DLC programs compared to RTP programs in all scenarios.

**Table 3:** Upper limit of customer participation for all program types and years.

Program	Year	Low-Flexibility	Flat and Mid-Flexibility	High-Flexibility
DLC	All	40%	62%	80%
RTP	All	44%	68%	88%
TOU	2025	5%	10%	100%
TOU	2030	5%	25%	100%
TOU	2035	5%	40%	100%
TOU	2040	5%	55%	100%
TOU	2045	5%	70%	100%
TOU	2050	5%	80%	100%

These bounds are assumed the same for LDVs and M/HDVs, and for new and recurring participants.

We model a wide range of upper limits on LDV TOU participation, from 5% to 100%. At the low end of the range, 5% participation as an upper limit reflects contemporary TOU participation rates that require customer awareness and motivation to manually avoid on-peak charging. At the high end of the range, pilots demonstrate a high potential for customer acceptance of TOU programs and automation that charges EVs during off-peak hours with simple communications and controls, increasing potential for perfect adherence to TOU time blocks within the constraints imposed by travel requirements. These lower and upper ranges are reflected in the Low-Flexibility and High-Flexibility scenarios, respectively. Flat and Mid-Flexibility scenario upper limits start at 10% and increase over time to 80% in 2050 as targeted marketing, customer familiarity, and automated charge scheduling increase participation.

For the fixed points on the curves, (x^*^, r^*^), the same surveys and pilots report DLC and RTP customer enrollment rates and associated incentive levels for LDV programs^6,8,9,17–20^. Participation rates and incentive levels are reported for M/HDVs in a utility program filing^13^. The mapping to fixed points by vehicle type, program type, scenario, and year is shown in Table 4, which, when combined with the $\overline{r}$ values listed in Table 3, enables calculation of *β*. This is sufficient to articulate response curves, which suggest incentive level ranges and how those map to participation rates. These were verified with subject matter experts and by comparison with the expectations of established EVMC programs^8,9,16,21,22^.

**Table 4:** Fixed points on the participation rate versus incentive level curves for recurring participants and new participants who do not need new chargers by vehicle type, program type, scenario, and year.

Vehicle Type	Program Type(s)	Scenario(s)	Year	r^*^	x^*^
LDV	DLC & RTP	Mid-Flex & Flat	2025	0.230	50
LDV	DLC & RTP	Mid-Flex & Flat	2030	0.308	50
LDV	DLC & RTP	Mid-Flex & Flat	2035	0.386	50
LDV	DLC & RTP	Mid-Flex & Flat	2040	0.464	50
LDV	DLC & RTP	Mid-Flex & Flat	2045	0.542	50
LDV	DLC & RTP	Mid-Flex & Flat	2050	0.619	50
LDV	DLC & RTP	High-Flex	2025	0.45	50
LDV	DLC & RTP	High-Flex	2030	0.50	40
LDV	DLC & RTP	High-Flex	2035	0.60	30
LDV	DLC & RTP	High-Flex	2040	0.70	20
LDV	DLC & RTP	High-Flex	2045	0.75	10
LDV	DLC & RTP	High-Flex	2050	0.79	0
^*a*^LDV	TOU	Mid-Flex	2025	0.01	5
^*a*^LDV	TOU	Mid-Flex	2030	0.02	5
^*a*^LDV	TOU	Mid-Flex	2035	0.03	5
^*a*^LDV	TOU	Mid-Flex	2040	0.05	5
^*a*^LDV	TOU	Mid-Flex	2045	0.07	5
^*a*^LDV	TOU	Mid-Flex	2050	0.09	5
^*a*^LDV	TOU	High-Flex & Flat	2025	0.18	85
^*a*^LDV	TOU	High-Flex & Flat	2030-2045	0.18	10
^*a*^LDV	TOU	High-Flex & Flat	2050	0.18	1
^*a*^LDV	TOU	Low-Flex	All	0.01	10
M/HDV	DLC & RTP	Mid-Flex	2025	0.230	500
M/HDV	DLC & RTP	Mid-Flex	2030	0.308	500
M/HDV	DLC & RTP	Mid-Flex	2035	0.386	500
M/HDV	DLC & RTP	Mid-Flex	2040	0.464	500
M/HDV	DLC & RTP	Mid-Flex	2045	0.542	500
M/HDV	DLC & RTP	Mid-Flex	2050	0.619	500
M/HDV	DLC & RTP	Flat	2025	0.230	900
M/HDV	DLC & RTP	Flat	2030	0.308	900
M/HDV	DLC & RTP	Flat	2035	0.386	900
M/HDV	DLC & RTP	Flat	2040	0.464	900
M/HDV	DLC & RTP	Flat	2045	0.542	900
M/HDV	DLC & RTP	Flat	2050	0.619	900
M/HDV	DLC & RTP	High-Flex	2025	0.45	150
M/HDV	DLC & RTP	High-Flex	2030	0.50	120
M/HDV	DLC & RTP	High-Flex	2035	0.60	90
M/HDV	DLC & RTP	High-Flex	2040	0.70	60
M/HDV	DLC & RTP	High-Flex	2045	0.75	30
M/HDV	DLC & RTP	High-Flex	2050	0.79	0
M/HDV	DLC & RTP	Low-Flex	All	0.073	810
M/HDV	TOU	Mid-Flex	2025	0.02	500
M/HDV	TOU	Mid-Flex	2030	0.03	500
M/HDV	TOU	Mid-Flex	2035	0.04	500
M/HDV	TOU	Mid-Flex	2040	0.05	500
M/HDV	TOU	Mid-Flex	2045	0.07	500
M/HDV	TOU	Mid-Flex	2050	0.09	500
M/HDV	TOU	High-Flex & Flat	2025	0.18	150
M/HDV	TOU	High-Flex & Flat	2030	0.18	120
M/HDV	TOU	High-Flex & Flat	2035	0.18	90
M/HDV	TOU	High-Flex & Flat	2040	0.18	60
M/HDV	TOU	High-Flex & Flat	2045	0.18	30
M/HDV	TOU	High-Flex & Flat	2050	0.18	0
M/HDV	TOU	Low-Flex	All	0.01	1760

^*a*^For this entry, x^*^ corresponds to the marketing costs per vehicle needed to achieve adherence rate r^*^.

In the case of new customers that require a new smart charger to enable participation, subject matter expert interviews indicate initial customer incentives will need to be higher to motivate the customer to purchase enabling hardware^7,23^. Many pilots and programs include such incentives, sometimes in the form of a smart charger rebate^9,24,25^. We assume this situation only applies to a fraction of LDV owners and only for DLC programs. The assumed increased incentive levels are listed in Table 5.

**Table 5:** Incentive level corresponding to r^*^ for new participants who need new chargers by vehicle type, program type, scenario, and year.

Vehicle Type	Program Type(s)	Scenario(s)	Year	x^*^ with install
LDV	DLC	Mid-Flex, High-Flex & Flat	All	300
LDV	DLC	Low-Flex	All	600

Only a fraction of LDV drivers interested in participating in DLC programs are assumed to need a higher initial incentive to help with purchasing a program-compatible charger. For all other vehicle and program types, participation versus incentive response is assumed to be the same across all new and recurring participants.

The proportion of customers requiring a new charger is expected to decrease over time as smart chargers become more prevalent and technology for smart charging becomes more ubiquitous. Based on work from Nicholas *et al*., in 2025, 19% of EV owners will require a new charger to participate in EVMC with private chargers, including first-time EV owners that are provided with a charger rebate to motivate EVMC program participation^26^. Some may not install a charger at home, whereas other customers that already own EVs could upgrade from L1 to L2 chargers. Price differences between smart chargers and chargers without two-way communications are decreasing. It is expected that, by 2050, most, if not all, Level 2 (L2) and direct current (DC) fast chargers will be equipped with smart charging support^1,17,27–30^. For this reason, the percentage of customers expected to require a new charger decreases to 0% by 2050, except in the Low-Flexibility scenario, in which 5% of customers are estimated to still require a new charger to participate in EVMC. It is assumed that M/HDV customers will already have access to a smart charger. The percentage of LDV customers requiring a new charger is shown in Table 6.

**Table 6:** Percentage of otherwise eligible LDV owners requiring a new EV charger to participate in smart charge management from 2025 to 2050.

Year	Low-Flexibility	Flat and Mid-Flexibility	High-Flexibility
2025	19%	19%	19%
2030	15%	10%	5%
2035	12%	5%	3%
2040	9%	3%	1%
2045	7%	2%	0%
2050	5%	0%	0%

**Fig. 3:**
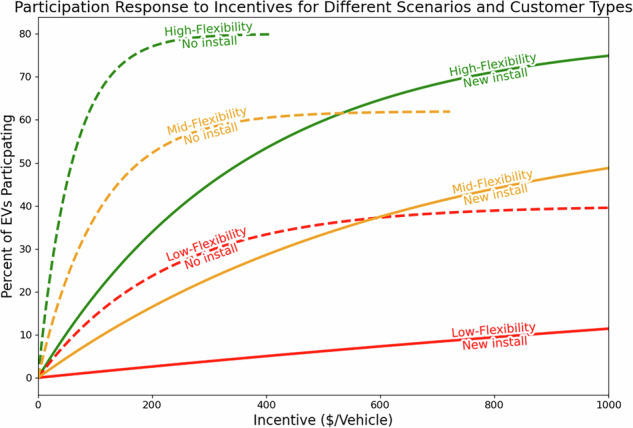
Customer enrollment as a response to annual incentives. These curves follow the form of Equation (1), in which upper limits of enrollment range from 40% to 80% (Table 3). The constant *β* is derived such that the resulting curve intersects data points found in literature (Tables 4 and 5). The different flexibility scenarios result in varied responses; customers’ need for additional equipment can have a larger impact on response than scenario.

These models result in many different curves describing customer response to incentives. Figure 3 shows six examples, all for LDV participation in DLC programs in 2025. In scenarios with greater levels of flexibility, both higher upper limits on participation and lower incentives required to reach the same level of participation as less-flexible scenarios result in more EVMC being accessible at lower cost. The figure shows that, as defined, the scenarios provide quite a wide range of possible response, demonstrating the influence of (x^*^, r^*^). A large response r^*^ to a lower incentive x^*^, as modeled in the high-flexibility scenario, results in the majority of eligible customers participate in a program for incentives under $100 per customer. In the least responsive case, large incentives approaching $1000 will still see fewer than 10% of eligible customers participating in this example program. Supply curve sensitivities to such tunable costs are represented in the different scenarios. It is also the case that the difference between requiring a new charger or not can be even larger than that of the different scenario assumptions.

In modeling participation as the percentage of EVs actively and fully participating in EVMC programs, no distinction is made between opt-in and opt-out programs, as sources indicate customer participation does not change significantly with program type^17^. Opt-out programs tend to enroll more customers, but, on average, customers in opt-out programs are less responsive to demand response program signals than customers in opt-in programs. Thus, on the whole, opt-in versus opt-out tends not to result in a difference in actual response.

#### Marketing Costs

Almost all programs are assumed to have marketing costs to inform EV drivers of the existence of EVMC programs and to encourage participation. The one exception is M/HDVs in the High-Flexibility scenario, for which we assume EVMC is handled as a standard part of commercial fleet operators working with their utility to install and power EVSE, such that no additional marketing costs are required^29^. Also, for almost all programs, marketing costs are assumed to be a fixed quantity per participating vehicle that varies by vehicle type, program type, scenario, and year. As explained previously, the one exception is LDV TOU programs, for which we assume choosing to spend more on marketing will result in higher levels of adherence to TOU rates (Equation (1), Tables 3, 4), participation in which is otherwise not incentivized.

The marketing costs for all programs other than LDV TOU are shown in Table 7. M/HDV marketing costs are estimated based on^13^. Marketing costs for LDV DLC and RTP programs are estimated based on^15,16^.

**Table 7:** Marketing costs for all vehicle types and programs except LDV TOU.

Vehicle Type	Program Type(s)	Scenario(s)	2025	2030	2035	2040	2045	2050
LDV	DLC & RTP	Mid-Flex & Flat	10	10	10	10	10	10
LDV	DLC & RTP	High-Flex	10	9	8	7	6	5
LDV	DLC & RTP	Low-Flex	20	20	20	20	20	20
M/HDV	DLC & RTP	Mid-Flex & Flat	80	40	20	10	8	5
M/HDV	TOU	High-Flex & Flat	0	0	0	0	0	0
M/HDV	DLC & RTP	High-Flex	0	0	0	0	0	0
M/HDV	DLC, RTP, TOU	Low-Flex	80	80	80	80	80	80

Values listed per year are in 2025-$ per participating vehicle. LDV TOU marketing costs are described previously with incentives for other programs because they are modeled as having a functional relationship with participation rates.

### Supply Curve Formulation

From all the costs described previously, we assemble supply curves for every combination of vehicle type (LDV or M/HDV), program type (DLC, RTP, or TOU), scenario, year, and customer type (new or recurring). In general, a supply curve describes how much resource can be obtained at what cost. In this dataset, the formulation of participation rates as a function of incentive levels, or as a function of marketing costs for LDV TOU programs, leads to supply curves that describe the cost per vehicle required to enroll a specified fraction *r* of eligible EVs in the given EVMC program.

There are slight variations in the costs included, and which cost varies based on participation rate, across all the supply curves. However, in all cases, there is exactly one term based on Equation (1), for which the cost *x* can be expressed as a function of participation rate *r* and parameters *β* and $\overline{r}$. In what follows, we express that cost *x* as ${C}_{\,{\rm{all}}}^{* }\left(r;\beta ,\overline{r}\right),$ where the superscript denotes the cost type (incentive or marketing) and the subscript “all” indicates the cost applies to both new and recurring customers. This cost can then be calculated as:

3 $${C}_{\,{\rm{all}}}^{\ast }(r;\beta ,\overline{r})=-\frac{1}{\beta }\cdot ln\left(1-\frac{r}{\overline{r}}\right),$$

where $\overline{r}$ and *β* correspond to the vehicle type, program type, scenario, and year of interest.

**Fig. 4:**
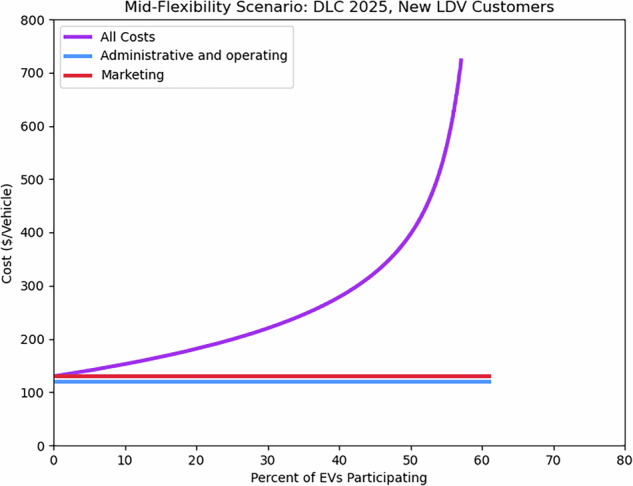
Example supply curve showing the per-vehicle cost of new customer enrollment in an LDV DLC program as a function of participation rate. The curve uses data for 2025 Mid-Flexibility assumptions. Per-vehicle marketing, administrative, and operating costs are assumed constant, whereas incentive level expresses the trade-off between total cost and amount of EVMC enabled.

The most complex supply curves correspond to new LDV participants enrolling in a DLC program. In this case, initial administrative costs for new participants apply, and there are two subpopulations of customers: those that need a new charger to participate, and those that do not. Thus, there are two supply curve expressions, one for each new customer subpopulation:

4 $$\begin{array}{c}{\rm{LDV\; DLC\; :}}\,\ \ {C}_{\,{\rm{new}},i}^{{\rm{total}}}(r)={C}_{{\rm{new}}}^{{\rm{admin}}}+{C}_{{\rm{all}}}^{{\rm{operating}}}+{C}_{{\rm{all}}}^{{\rm{marketing}}}+{C}_{{\rm{all,}}\,{\rm{i}}}^{{\rm{incentive}}}(r;{\beta }_{i},\overline{r}),\\ \,i\in \{{\rm{no\; install}},{\rm{with\; install}}\}\end{array}$$

that must be blended together (which is done by discretizing, merging, and sorting by ascending total costs) to understand the supply curve as a whole. For example, in 2025, 19% of new LDV DLC participants are expected to need a new charger to participate (Table 6), and under the Mid-Flexibility scenario, those customers are expected to need a $300 incentive (rather than the $50 incentive required by customers that do not need a new charger) to reach a 23% enrollment rate (Tables 4 and 5). Interweaving the supply curves of both types of new participants yields the supply curve shown in Fig. 4.

For all potential participants, initial administrative costs apply: $60 per vehicle, program operating costs of $60 per vehicle, and marketing costs of $10 per vehicle. The response to incentives differs across the 19% of potential participants that need a new charger to participate and the 81% that do not. In both cases, the maximum participation rate is assumed to be 62%, which the supply curve approaches in the limit of an infinite incentive.

The structures of the other supply curves in the dataset are similar. Specifically, they are:

5 $${\rm{LDV\; TOU:}}\,{C}_{{\rm{new}}}^{{\rm{total}}}(r)={C}_{{\rm{new}}}^{{\rm{admin}}}+{C}_{{\rm{all}}}^{{\rm{operating}}}+{C}_{{\rm{all}}}^{{\rm{marketing}}}(r;\beta ,\overline{r})$$

6 $${C}_{\,{\rm{recurring}}}^{{\rm{total}}}(r)={C}_{{\rm{all}}}^{{\rm{operating}}}+{C}_{{\rm{all}}}^{{\rm{marketing}}}(r;\beta ,\overline{r})$$

7 $${\rm{All\; Others:}}\,{C}_{{\rm{new}}}^{{\rm{total}}}(r)={C}_{{\rm{new}}}^{{\rm{admin}}}+{C}_{{\rm{all}}}^{{\rm{operating}}}+{C}_{{\rm{all}}}^{{\rm{marketing}}}+{C}_{{\rm{all}}}^{{\rm{incentive}}}(r;\beta ,\overline{r})$$

8 $${C}_{\,{\rm{recurring}}}^{{\rm{total}}}(r)={C}_{{\rm{all}}}^{{\rm{operating}}}+{C}_{{\rm{all}}}^{{\rm{marketing}}}+{C}_{{\rm{all}}}^{{\rm{incentive}}}(r;\beta ,\overline{r})$$

with High-Flexibility M/HDV marketing costs equal to zero for all program types and High-Flexibility M/HDV TOU initial administrative costs also equal to zero.

Note that, within a given supply curve, we do not model any per-vehicle costs decreasing as participation rates increase, which could be the case if economies of scale are realized within the time frame of 1 year. Current data sources do not describe such within-year economies of scale but do support the two types of costs efficiencies present in the dataset. Specifically, it costs less to continue participation year on year than to enroll new participants, and we model year-on-year cost declines for some cost categories and under some scenario conditions.

### Cost Category Influences on Supply Curves

Ranges in the various cost categories and their influence on supply curves are represented through the scenario narratives described before. Here, supply curve sensitivity to those ranges and within the scenarios is explored. Different participation responses to tunable costs such as customer incentives produce a wide range of per vehicle costs, a sample of which is shown in Fig. 3. The variation in all flat costs that appear for LDVs and M/HDVs in different programs is shown in Table 8.

**Table 8:** Ranges of flat costs associate with different vehicle types and programs in 2025.

Vehicle Type	Program Type(s)	Customer Type	Minimum	Maximum
LDV	TOU	Recurring	0	60
LDV	TOU	New	10	180
LDV	DLC & RTP	Recurring	94	380
LDV	DLC & RTP	New	34	140
M/HDV	TOU	Recurring	0	120
M/HDV	TOU	New	250	370
M/HDV	DLC & RTP	Recurring	250	250
M/HDV	DLC & RTP	New	500	500

These cost categories include flat marketing costs, program operating costs, and initial administrative costs. Values listed per year are in 2025-$ per participating vehicle. There is no variability in M/HDV flat costs for RTP and DLC programs, as the dataset is very limited for such cases and no variability was found in the data.

**Fig. 5:**
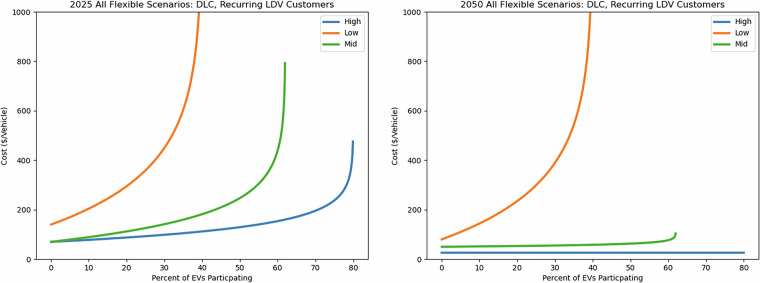
Example supply curves showing the per-vehicle cost of recurring customer enrollment in an LDV DLC program in 2025 (left) and 2050 (right) as a function of participation rate.

As flat costs per vehicle increase, supply curves reflect greater expense to the utility at all levels of participation. Figure 5 combines all cost categories to show a range of supply curves resulting from the highest and lowest costs for all cost categories for LDVs participating in DLC programs. The 2025 case represents the greatest range in flat costs, resulting in a low-flexibility scenario where customers willing to participate for a minimal incentive are $286 more costly than in a high-flexibility scenario with lower flat costs. To achieve higher participation rates, increases in necessary incentives dominate the cost differences between scenarios. Figure 5 shows this range in 2025 as well as the trajectory of supply curves into 2050.

**Table 9:** Sources for cost data, customer participation response to incentives and marketing, and other assumptions informing EVMC supply curves.

Category	Cost data, incentive response, enrollment, or assumption	Source
Participation versus incentives	33%–74% participation from $300 initial incentive	^6^
	45% participation from $300 initial incentive (with a new charger required)	^9,22^
	45% participation from $50 annual incentive (no new charger required)	^9,22^
	Pilot with 85% enrollment for $65 and free or discounted charge point	^9^
	7.3% participation for $45 annual incentive	^8^
Incentive ranges	$100-$150 per vehicle per year	^21^
	$450 charger rebate	^9^
	$1760 for commercial EVs	^13^
Similar participation for RTP & DLC	No difference in participation is observed due to EVMC program type	^17^
Operations and administrative costs	Recurring operating costs are $14-$20 per year in the residential sector and $20 per year in the commercial sector	^15^
	Initial administrative costs: $50 per residential customer and $50-$350 per commercial customer. Recurring administrative costs: $10 per customer per year	^15^
	Commercial recurring annual program costs of $250 per EV	^13^
	Program administrative costs, utility administrative costs, and M&V total $59 per EV	^16^
Participation versus marketing	$85 per vehicle, resulting in 12% participation (TOU for LDV)	^34^
	Customer participation varies with marketing	^35^
	Marketing drives greater customer enrollment in TOU programs	^20^
Marketing	Marketing is $5-$10 per site per year	^15^
	Marketing costs are initially $80 per commercial EV and drop to $0 in forecast years	^13^
	Initial marketing costs of $44 per EV in first year, $9.18 by 2027	^16^
Hardware	19% of all EV owners will have a L1 or L2 charger at home (or will not charge their EV at home) that cannot be programmed to participate in smart charging programs	^26^
	By 2030, more than 95% of all public charging sites will be capable of participating in smart charging	^1^
	A WiFi or cellular connection for home chargers is required in the United Kingdom	^27^
	Costs of public chargers to enable EVMC is minimal	^28^
	The National Electric Vehicle Infrastructure Formula Program requires that smart charging be supported	^30^
	Smart charging enablement occurs with EVSE site install for commercial sites/fleet vehicles	^29^
	All chargers will be networked by 2050	^17^
Upper limits	Survey of demand response studies indicates a potential of 70%–100% enrollment of eligible customers for EV programs	^17^
	27% of survey participants were willing to accept DLC, 30% responded “maybe,” and 43% responded “no”	^18^
	Survey data on participation versus incentives show 12%–20% of EV owners would not participate in demand response programs for $750 to $1000 per year (the survey did not ask about incentives over $1000)	^6^
	Technical issues and opt-outs resulted in 13–41% of all plugged in vehicles did not participate in a demand response event	^8^
	Participation in “utility-controlled charging” could be 63%–78% of customers	^19^
	Where available, enrollment of eligible customers in TOU ranges from 7%–30%	^35^
Participation over time	Automation technology increases customer participation	^9^

Certain data were excluded, including participation reported as a percentage of the participants in a given study or pilot project (values that are always near 100%), participation reported as a percentage of limited available chargers provided as incentives, or incentives that were not monetary, such as coordination with Public Safety Power Shutoff events^9,36^.

The low-flexibility scenario shows relatively little change in costs per vehicle from 2025 to 2050, while the mid- and high-flexibility scenarios decrease at all levels of participation.

### Supply Curve Changes From 2025 to 2050

**Fig. 6:**
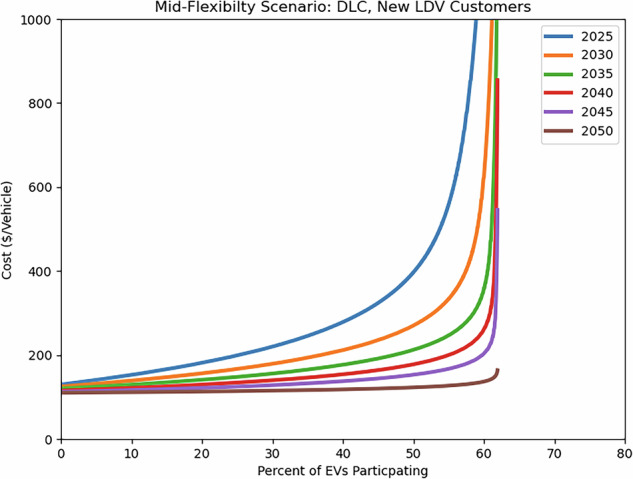
Example supply curves showing cost decreases over time for a single vehicle type, program type, customer type, and scenario, specifically LDVs newly enrolling in a DLC program under Mid-Flexibility conditions. In this example, incentives and program operating costs decline over time, while initial administration costs, marketing costs, and the upper limit of participation are held constant.

In most cases, costs per vehicle decrease over time as technology improves, customers become more knowledgeable, and program design successfully targets more customers. Figure 6 shows this decreased cost between 2025 and 2050 in a Mid-Flexibility scenario. Similar trends are observed across all programs and across all scenarios other than Low-Flexibility, in which costs and achievable participation rates are assumed to change little over time.

### Impact of Dispatch Mechanism

**Fig. 7:**
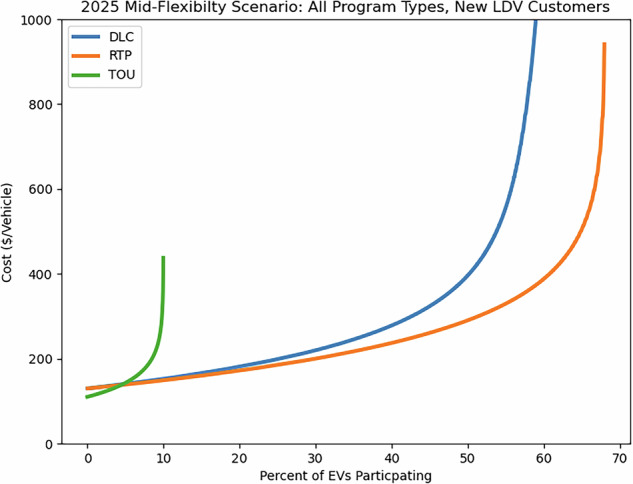
Example set of curves for the same scenario and variables with different programs. This instance shows the percentage of participating LDVs eligible for each program in a Mid-Flexibility scenario. DLC is potentially the most expensive program per vehicle at higher participation rates, while RTP has the potential for the highest levels of customer participation. TOU participation in 2025 is limited, resulting in an asymptote at 10% participation. This reflects participation levels found in the literature^20^.

Three supply curves for the different programs and all other variables held constant are shown in Fig. 7.

Comparisons between programs vary over time, scenario, and vehicle type, but in all cases, DLC can be the most costly to implement, particularly at higher participation levels. DLC programs provide utilities with the greatest degree of control over EV charging but require additional communications and more complex operations. Although some program costs may be similar to those of RTP, the overall costs for DLC and RTP programs are different. With fewer communication requirements, simpler controls, and possibly more customer acceptance, RTP costs are consistently lower than that of DLC for the same level of participation. At low rates of participation, TOU is generally less expensive than DLC and RTP, but, as described in above, contemporary TOU tends to have low participation because of a lack of automation (or the use thereof) to automatically schedule charging. Thus, in the 2025 data shown in Fig. 7, participation is constrained to the limited levels found in the literature^20^. This increases over time in all but the Low-Flexibility scenario.

## Data Records

The data are available on Zenodo^31^. Additionally, the data and code used to generate them are available on GitHub^32^. The summary dataset consists of a .csv file of total per-vehicle costs for increasing percentages of customer enrollment in 1% increments. Each row lists costs, upper participation limit, and participation rate for a given incentive, for a given program, scenario, vehicle type, and year.

## Technical Validation

This dataset is established with cost data from utility programs and pilots, prior research that established costs for demand-side resources, customer surveys, and subject matter expert interviews^7,23,33^. Final results were vetted by subject matter experts. Table 9 shows the range of costs identified and sources that informed assumptions around customer responses to incentives and marketing, as well as sources for assumptions around cost category inclusion or exclusion for different program types and forecasting cost and participation changes in future years.

## Usage Notes

The forward-looking supply curves presented here can be used by grid planners or any party examining the expected costs of EVMC programs. These supply curves can be used to estimate the per-vehicle costs of participation in EVMC at specified adoption levels: for example, when 20% of eligible LDV owners in 2025 enroll in DLC, the cost in a mid-flexibility scenario is $112 per vehicle. This can be converted to kilowatts (kW) when combined with a per-vehicle capacity estimate, which will be less than or equal to the charger rating. These supply curves allow the user to select appropriate kW-per-vehicle values reflecting the region, time, and EV adoption.

Although the different scenarios provide bounds to assist users in assessing EVMC adoption potential, there are data limitations because of the novelty of this technology, especially for electric M/HDVs, which are in very early stages of adoption. Many sources for data relevant to formulating supply curves include surveys and pilots, rather than long-term results of programs rolled out to a utility’s entire eligible customer base. There will also be regional differences resulting from regulatory and policy implementations. These differences can impact interrelated phenomena, such as the amenability of customers and the availability of smart-charge-enabled EVSE. For any use case, regional variables or implementation specifics can inform user choices in selecting the most appropriate supply curves (e.g., by scenario and program).

## Data Availability

The data are available on Zenodo: https://zenodo.org/records/18764256. This includes a .csv file of total per-vehicle costs for increasing percentages of customer enrollment in 1% increments and a .csv file of the scenario variables used to generate the supply curves.

## Code availability

The code to generate and query EVMC supply curves is available in a public repository: https://github.com/dsgrid/evmc-supply-curves. The repository includes installation, usage and other instructions, as well as an example Jupyter notebook.

## References

[1] Wood, E. *et al*. The 2030 National Charging Network: Estimating U.S. Light-Duty Demand for Electric Vehicle Charging Infrastructure. Tech. Rep. https://docs.nrel.gov/docs/fy23osti/85654.pdf (2023).

[2] Global EV Outlook 2024. Tech. Rep., Paris https://www.iea.org/reports/global-ev-outlook-2024 (2024) .

[3] International Energy Agency. Trends in electric cars. Online report https://www.iea.org/reports/global-ev-outlook-2024/trends-in-electric-cars (2024).

[4] Anwar, M. B. *et al*. Assessing the value of electric vehicle managed charging: a review of methodologies and results. *Energy & Environmental Science* **15**, 466–498 (2022).

[5] Hale, E. *et al*. Electric Vehicle Managed Charging: Forward-Looking Estimates of Bulk Power System Value. Technical Report NREL/TP-6A40-83404, National Renewable Energy Laboratory (NREL), Golden, CO (United States) https://www.osti.gov/biblio/1890139 (2022).

[6] Wong, S. D., Shaheen, S. A., Martin, E. & Uyeki, R. Do incentives make a difference? Understanding smart charging program adoption for electric vehicles. *Transportation Research Part C: Emerging Technologies* **151**, 104123 (2023).

[7] Polis, H. & Buhr, T. Personal communication. opinion Dynamics (2024).

[8] National Grid EV and PHEV Demand Response Evaluation. Tech. Rep., Guidehouse Inc https://ma-eeac.org/wp-content/uploads/MA21-DR04-E-Res-EV-National-Grid-EV-and-PHEV-Demand-Response-Evaluation-Report-2022-07-11-FINAL-wInfographic.pdf (2022).

[9] Blair, B., Dougherty, C. & Fitzgerald, G. Managed Charging Programs: Maximizing Customer Satisfaction and Grid Benefits. Tech. Rep., Smart Electric Power Alliance https://sepapower.org/resource/managed-charging-programs-maximizing-customer-satisfaction-and-grid-benefits/ (2023).

[10] Gerke, B. F. *et al*. The California Demand Response Potential Study, Phase 4: Report on Shed and Shift Resources Through 2050. Tech. Rep., Lawrence Berkeley National Lab. (LBNL), Berkeley, CA (United States) https://buildings.lbl.gov/download-page-phase-4-final-report-shed-and-shift-resources-through-2050 (2024).

[11] Bengtson, N., Sergici, S., Eamonn, U. & Hagerty, M. Designing Effective EV Managed Charging Programs https://www.brattle.com/wp-content/uploads/2021/10/Designing-Effective-EV-Managed-Charging-Programs.pdf.

[12] Hledik, R., Peters, K. & Edelman, S. California’s Virtual Power Potential: How Five Consumer Technologies Could Improve the State’s Energy Affordability, Volume II: Technical Appendix. Tech. Rep., The Brattle Group https://www.brattle.com/wp-content/uploads/2024/04/Californias-Virtual-Power-Potential-How-Five-Consumer-Technologies-Could-Improve-the-States-Energy-Affordability-Technical-Appendix.pdf (2024).

[13] Massachusetts Electric Company and Nantucket Electric Company each d/b/a National Grid. Direct Pre-Filed Testimony of the Electric Vehicle Program Panel: Exhibit NG-EVPP-1. Tech. Rep. D.P.U. 21-91, https://eeaonline.eea.state.ma.us/dpu/fileroom/#/dockets/docket/11988 (2021).

[14] Getting ahead of the EV tipping point. Tech. Rep., https://www.aes.com/ev-tipping-point (2024).

[15] Alstone, P. *et al*. 2025 California Demand Response Potential Study - Charting California’s Demand Response Future. Final Report on Phase 2 Results. Tech. Rep. 1421800 http://www.osti.gov/servlets/purl/1421800/ (2017).

[16] Baltimore Gas and Electric Company. Smart Charge Management Program Proposal of Baltimore Gas and Electric Company. Tech. Rep. Case No. 9478, Mail Log No. 310052, Public Service Commission of Maryland https://webpscxb.psc.state.md.us/DMS/maillogsearch (2024).

[17] Parrish, B., Gross, R. & Heptonstall, P. On demand: Can demand response live up to expectations in managing electricity systems? *Energy Research & Social Science* **51**, 107–118 (2019).

[18] Yilmaz, S. *et al*. Analysis of demand-side response preferences regarding electricity tariffs and direct load control: Key findings from a Swiss survey. *Energy* **212**, 118712 (2020).

[19] Bailey, J. & Axsen, J. Anticipating PEV buyers’ acceptance of utility controlled charging. *Transportation Research Part A: Policy and Practice* **82**, 29–46 (2015).

[20] Szinai, J. K., Sheppard, C. J., Abhyankar, N. & Gopal, A. R. Reduced grid operating costs and renewable energy curtailment with electric vehicle charge management. *Energy Policy* **136**, 111051 (2020).

[21] Grunkmeyer, B. Personal Communication. FlexCharging (2024).

[22] Opinion Dynamics PG&E EV Automated Demand Response Study Report. Tech. Rep., Opinion Dynamics https://opiniondynamics.com/wp-content/uploads/2022/03/PGE-EV-ADR-Study-Report-3-16.pdf (2022).

[23] Dougherty, C. & Fitzgerald, G. Personal communication. smart Electric Power Alliance (2024).

[24] Cook, J., Churchwell, C. & George, S. Final Evaluation for San Diego Gas & Electric’s Plug-in Electric Vehicle TOU Pricing and Technology Study. Tech. Rep., https://www.sdge.com/sites/default/files/SDGE%20EV%20%20Pricing%20%26%20Tech%20Study.pdf (2014).

[25] Brittany Blair, Garrett Fitzgerald & Carolyn Dougherty. The State of Managed Charging in 2021. Tech. Rep., Smart Electric Power Alliance (SEPA) https://sepapower.org/resource/the-state-of-managed-charging-in-2021/ (2021).

[26] Nicholas, M. Estimating electric vehicle charginginfrastructure costs across major U.S. metropolitan areas https://theicct.org/publication/estimating-electric-vehicle-charging-infrastructure-costs-across-major-u-s-metropolitan-areas/.

[27] Complying with the Electric Vehicles (Smart Charge Points) Regulations 2021: Guidance for sellers of electric vehicle charge points in Great Britain https://assets.publishing.service.gov.uk/media/628ce214e90e071f653a494a/Guide-to-evscp-regulations-2021-V2.1.pdf (2022).

[28] Crisostomo, N. Electric Vehicle Charging Load .

[29] Workplace Fleet Charging Case Study: In partnership with Sacramento Municipal Utility District (SMUD). Tech. Rep. https://f.hubspotusercontent40.net/hubfs/4962241/_Commercial%20Content/Case%20Studies/091520-SMUDCaseStudy.pdf?hstc=136157294.853ad149a379a7554db06db2d0fda1d5.1647370526237.1647370526237.1647370526237.1.

[30] National Electric Vehicle Infrastructure Standards and Requirements https://www.govinfo.gov/content/pkg/FR-2023-02-28/pdf/2023-03500.pdf (2023).

[31] Reiko Matsuda-Dunn, Elaine Hale, Gabriel Konar-Steenberg & Luke Lavin. Bounding the costs of electric vehicle managed charging-supply curves for scenarios from 2025 to 2050 10.5281/zenodo.18764256 (2025). 10.1038/s41597-026-07008-6 PMC13111587 41813704

[32] Reiko Matsuda-Dunn, Elaine Hale, Gabriel Konar-Steenberg & Luke Lavin. dsgrid/evmc-supply-curves https://github.com/dsgrid/evmc-supply-curves (2025). 10.1038/s41597-026-07008-6 PMC13111587 41813704

[33] Parkinson, K. & LeBlanc, B. Personal communication. peak Load Management Alliance (2024).

[34] Potter, J. M., George, S. S. & Jimenez, L. R. SmartPricing Options Final Evaluation: The final report on pilot design, implementation, and evaluation of the Sacramento Municipal Utility District’s Consumer Behavior Study. Tech. Rep., https://www.energy.gov/sites/prod/files/2016/12/f34/CBS_Final_Program_Impact_Report_Draft_20161101_0.pdf (2014).

[35] Myers, E., Hargrave, J., Farinas, R., Hledik, R. & Burke, L. Residential Electric Vehicle Rates That Work Attributes That Increase Enrollment. Tech. Rep., Smart Electric Power Alliance https://sepapower.org/resource/residential-electric-vehicle-time-varying-rates-that-work-attributes-that-increase-enrollment/ (2019).

[36] Phillips, R. & Ward, J. Project Lessons from Trial Recruitment: Customer-Led Network Revolution Trials. Tech. Rep. https://sustainabilityfirst.org.uk/wp-content/uploads/2013/07/CLNR-Lessons-from-Trial-Recruitment-Sustainability-First-paper-24-July-2013.pdf (2013).

